# Traumatic Brain Injury and Related Antisocial Behavioral Outcomes: A Systematic Review

**DOI:** 10.3390/medicina59081377

**Published:** 2023-07-27

**Authors:** Giuseppa Maresca, Viviana Lo Buono, Anna Anselmo, Davide Cardile, Caterina Formica, Desiree Latella, Angelo Quartarone, Francesco Corallo

**Affiliations:** 1 IRCCS Centro Neurolesi Bonino-Pulejo, S.S. 113 Via Palermo, C.da Casazza, 98124 Messina, Italyviviana.lobuono@irccsme.it (V.L.B.); caterina.formica@irccsme.it (C.F.); desiree.latella@irccsme.it (D.L.); francesco.corallo@irccsme.it (F.C.); 2 University of Torino, 10124 Torino, Italy; anna.anselmo23@gmail.com

**Keywords:** traumatic brain injury (TBI), aggression, brain imaging, criminal behavior, violence, forensic psychiatry, juvenile offenders, psychiatric comorbidity

## Abstract

*Background and Objectives*: Higher level of aggression and antisocial behavior have been found in the period following head trauma. These changes are attributable to specific brain alterations that generally involved frontal lobe, insula and limbic system. A descriptive review was conducted on the specificity of aggressive behavior in relation to traumatic brain injury by evaluating numerous variables, focusing on age at the time of trauma and neuroimaging studies. *Materials and Methods*: We searched on PubMed and the Web of Science databases to screen references of included studies and review articles for additional citations. From an initial 738 publications, only 27 met the search criteria of describing the relationship between aggression, brain alterations and traumatic brain injury. *Results*: These findings showed that traumatic brain injury (TBI) is related to changes in behavior, personality and mood. *Conclusions*: The development of aggressive and criminal behavior is associated with multiple factors, including the etiology of injury, environmental, psychosocial and personality factors and age at the time of trauma.

## 1. Introduction

Traumatic brain injury (TBI) has been suggested as a factor that leads to aggressive behavior through sequelae such as behavioral dysregulation or impulsiveness, causing the subjects to become involved in violent and criminal behavior [1,2]. A higher level of aggression has been found in the acute period following head trauma [3,4]. In postacute phases, a significant range of psychiatric disorders occurs, especially in term of antisocial behaviors; both violent and non-violent [5]. “Antisocial behavior” refers to activities on the most severe end of the spectrum of socially unacceptable behavior, such as rule breaking, delinquency, nuisance behavior, vandalism and physical and verbal aggression [6,7]. The correlation between TBI and changes in mood, personality and behavior has been demonstrated, as well as the possible consequences [8].

The implications of TBI can involve physical, cognitive, emotional or behavioral disorders which significantly compromise the individual’s independence, interpersonal relationships and work [9,10].

Many investigations have made links between brain injury and aggression by focusing on estimates of the frequency of aggressive behaviors during the acute period after TBI [3]. A history of TBI is associated with increased aggressiveness, drug and alcohol use, cognitive functioning disorders and antisocial behavior as an outcome of mental health disorders [11,12]. These factors can contribute to offending behavior and can make community placement more difficult. Indeed, patients with a history of head injury and mental illness often have poor social skills and are ill-equipped to cope with community living, requiring continued support [13]. TBI survivors may have particular behaviors that predispose them to certain acts of maladjustment. Among prisoners in particular, a prevalence of stories of head trauma has been found, which has been linked to the perpetration of criminal and violent acts [14].

Based on these data, the present literature review aims to address the following issues:

- How TBI is related to aggression and antisocial behavior;
- The role of biopsychosocial predisposing factors;
- Neuroanatomical correlates of criminal and aggressive behavior;
- The correlation between younger TBI and psychiatric comorbidity.

## 2. Materials and Methods

### 2.1. Search Strategy

A review of currently published studies was performed for articles on the aggressive behavior in relation to traumatic brain injury by evaluating numerous variables, focusing on age at the time of trauma and neuroimaging studies. The literature search was conducted via PubMed and Web of Science, and it was carried out for articles published from 2003 onward using the following search keywords terms: (“brain injuries, traumatic” [MeSH Terms] OR (“brain” [All Fields] AND “injuries” [All Fields] AND “traumatic” [All Fields]) OR “traumatic brain injuries” [All Fields] OR (“traumatic” [All Fields] AND “brain” [All Fields] AND “injury” [All Fields]) OR “traumatic brain injury” [All Fields]) AND (“aggression” [MeSH Terms] OR “aggression” [All Fields]).

### 2.2. Inclusion Criteria

A study was included if it described or investigated patients with a history of traumatic brain injury from any cause and any degree of severity. Only articles written in English were included in the review.

### 2.3. Exclusion Criteria

A study was excluded if there was a lack of patients with traumatic brain injury. Systematic, integrative or narrative reviews were also excluded, although their reference lists were reviewed and included if appropriate. All articles written in languages other than English were excluded. Studies published before 2003 were excluded from this research.

## 3. Results

**Figure 1:**
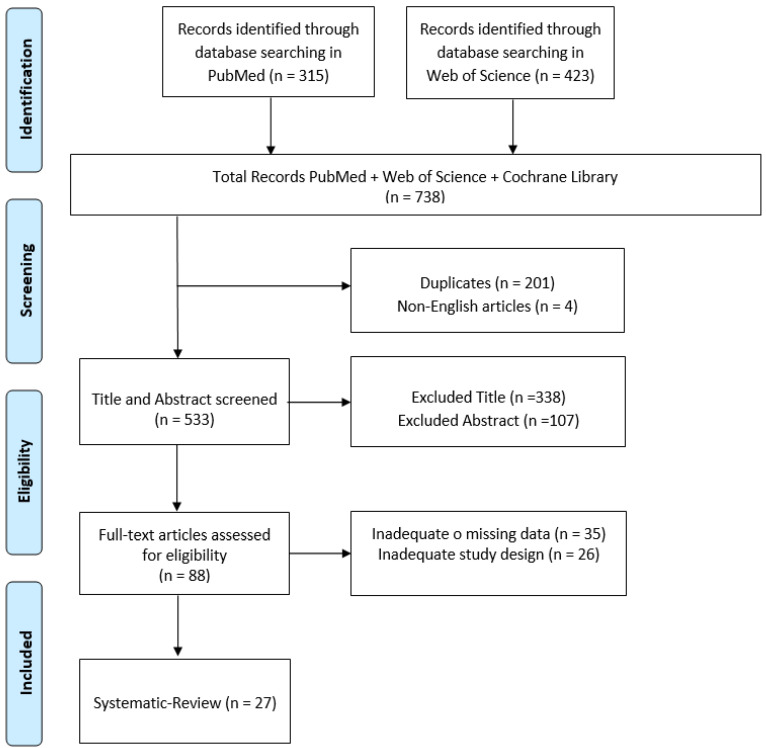
PRISMA flow chart for the current review.

The initial electronic data search yielded a total of 738 potentially relevant studies on PubMed and Web of Science. After removing all duplicates from the initial list, the articles were carefully evaluated based on their title, abstract and text. Implementation of these procedures resulted in only 27 eligible papers for inclusion of the initial 738 articles within the search (Figure 1). All the included studies are reported in Table 1.

### 3.1. TBI, Aggression and Antisocial Behavior

TBI is a risk factor for offensive behavior towards others, due to emotional lability and trauma-related irritability [27]. Colantonio et al. [14] demonstrated the high prevalence of TBI histories among prisoners related to criminal and violent acts. Specifically, the authors found that 60.25% of adult offenders had experienced at least one TBI. In 2015, Schofield et al. [1] hypothesized a relationship between traumatic brain damage and the subsequent behavioral dysregulation and impulsiveness that leads to aggressiveness and involvement in criminal acts. They used whole-population data linkage to identify individuals born in Western Australia (WA) between 1980 and 1985 inclusive who had attended a hospital in WA with recorded evidence of a TBI. Of these, they randomly selected three individuals of the same sex and year of birth born in WA who had never been admitted to the hospital for TBI. The results of this retrospective cohort data linkage study are consistent with a modest increase in the risk of offending, including violent offending, following a hospital-documented TBI. A younger age at the time of the injury is an important predictor of aggression following head trauma. Indeed, individuals who were younger at the time of the trauma were more likely to become aggressive. Baguley et al. [15] showed that individuals who were younger at the time of injury or depressed following rehabilitation were most likely to become aggressive. The study was conducted on 228 patients (71.5% of the original sample). Aggression was measured using the The Overt Aggression Scale (OAS). The OAS measures both global aggression and four specific subtypes of aggression (verbal, physical toward objects, physical toward self and physical toward others). 

The main findings of the study were that aggression rates were approximately 25% after TBI, and that higher depression rates and a younger age at the time of injury were the most significant predictors of aggression after TBI [11]. In 2017, Gordon [28] showed a correlation between TBI and subsequent crime, emphasizing the need for timely identification of TBI in young people at risk, such as adolescents using substances or those with antisocial behavior. More than 4000 juvenile delinquents have been screened for TBI, and the results revealed that TBI was common in these individuals, with 21.9% of the state sample and 41.3% of the county sample meeting the criteria for at least one TBI. A remarkable result of this study is that a TBI preceded a criminal offence in most of the state (56.5%) and county (78.5%) samples of participants with a TBI. Although this finding does not directly imply the causality of the crime, it provides strong evidence that TBIs and their subsequent effects on cognition and behavioral regulation changes are significant factors contributing to arrest that are separated from other premorbid risk factors. Jackson et al. [30] found further evidence suggesting that head trauma in childhood is associated with increased arrest rates later in life. Children who suffered head trauma had 1.5 times the arrest rate in life, and those who suffered severe head trauma had more than twice the arrest rate in childhood compared to those who did not suffer head trauma [30]. Given the incidence and persistence of irritability, anger and aggression after TBI, Miles et al. [32] highlighted importance of educating providers and family on the different ways and times that these behaviors can manifest.

### 3.2. The Role of Biopsychosocial Predisposing Factors

Aggression following TBI is associated with multiple biological and psychosocial factors, including major depression, substance abuse, disconnection from family relationships and poor work performance [31]. Tateno [31] highlighted the incidence of related factors by focusing on disorders such as major depression and substance abuse that increased the generation of violent behavior. The study group consisted of 89 consecutive patients with closed head injuries admitted to the University of Iowa Hospitals and Clinics (n = 58) and the Iowa Methodist Medical Center in Des Moines, Iowa (n = 31). Of the 89 patients who suffered a traumatic brain injury, 30 of them (33.7%) met the aforementioned criteria for the presence of significant aggressive behavior during the first six months after the traumatic episode. The remaining 59 patients (66.3%) constituted the nonaggressive group.

The frequency of a history of alcohol and substance abuse was significantly higher among aggressive patients than among non-aggressive patients. Moreover, impaired psychosocial functioning after trauma is a factor associated with aggression [35]. TBI sequelae can impair the abilities of individuals, causing poor anger control, reduced judgment and memory dysfunction [22]. Indeed, head trauma can compromise an individual’s ability to suppress aggressive impulses, particularly in the context of excessive alcohol use, thus increasing the possibility of violent crime and criminal convictions [38].

Differences in symptom development after TBI are also influenced by social factors, such as trauma treatment and social support [39]. Temperament has been indicated as the principal predictive factor of violence in the relationship between TBI and subsequent violence [16]. In a 2018 study, Darby [18] stressed the importance of certain factors that increase the evolution of aggressive behavior following head trauma. The factors identified included genetic factors, age at the time of trauma and environmental factors. Similarly, in 2010, Williams [17] highlighted a correlation between the high risk of head trauma in youth and the risk of post-trauma behavior offending disorders. These studies have limitations, and an examination of the more specific relationship between head trauma and aggression, and between head trauma in young people and future criminal behavior, is needed. Despite these limitations, they found a correlation between head trauma (TBI) and neurobehavioral consequences, which related to the incidence of other factors such as the severity of the trauma, age at the time of the trauma, social and environmental factors and a previous history of alcohol and drug abuse. These factors correlated to an escalation in the development of behavioral disorders related to aggression, which in some cases resulted in criminal and violent acts.

These studies have shown a correlation between TBI and subsequent crime, demonstrating the importance of timely identification of TBIs in young people at risk, such as adolescents using substances or exhibiting antisocial behavior. Providing the right tools for assessment and enabling cognitive intervention before the neurobehavioral consequences of trauma contribute to crime is crucial [28].

In summary, aggression following TBIs is associated with multiple biological and psychosocial factors, including major depression, substance abuse and impaired social function, as well as the presence of brain injury involving the frontal lobe. Understanding the clinical and demographic factors that predispose people to aggressive behavior as a result of TBI can be the key to identifying predictive factors and can help us to perform early intervention.

### 3.3. Neuroanatomical Correlates of Criminal and Aggressive Behavior

Neuroimaging studies have identified the areas most affected by TBI that may be responsible for deviant behavioral changes [19]. For instance, in one study, 64.5% of murderers were found to have frontal lobe dysfunction on examination, and approximately 47% had reportedly abnormal neuroimaging [34]. The brain regions that could be involved in aggressive behavior are the amygdala and insula, while prefrontal areas such as the orbitofrontal cortex (OFC) serve to inhibit aggression. Damage to these regions can compromise the balance of this starting/inhibition system and influence behavior [24].

Frontal lobe dysfunction has been used to explain the actions of some people charged with or convicted of violent crimes who apparently failed to inhibit their impulsive behaviors [40]. Elevated aggression was also associated with reduced white matter integrity in the corpus callosum, which is known to play an important role in multiple affective processes [26].

The limbic system and the frontal and temporal lobes are the areas of the brain that are most involved in the production of violent behavior. The structure most involved in violent behavior is the amygdala. A lack of empathy and remorse have been correlated with bilateral damage to the amygdala, while violence has been correlated with abnormal electrical activity in the amygdala [41]. Functional neuroimaging has shown the existence of a neurological basis in aggressive or violent behavior, as well as psychosocial factors. In particular, aggressiveness, impulsiveness and violence are associated with prefrontal and subcortical brain areas, which are the centers responsible for regulating emotions [42]. Moreover, the frontal lobes are responsible not only for executive functioning and social behavior, but also for moral decision making, value-based decision-making and theory of mind [19,20]. The finding of frontal lobe involvement in aggressive behavior highlights the importance of neuroimaging in finding a correlation between brain areas damaged by trauma and resulting deviant behavior.

In 2016, Epstein et al. [24] found that the brain regions thought to contribute to aggression include the amygdala and insula, while prefrontal areas, including the orbitofrontal cortex (OFC), serve to inhibit aggression. Damage or metabolic changes in these regions can influence the balance of this initiation/inhibition system and subsequently influence behavior. In 2010, Žarković et al. [41] attributed the production of violent behavior to the limbic system and the frontal and temporal lobes. The structure most involved in violent behavior in this research was the amygdala. A lack of empathy and remorse was correlated with bilateral damage to the amygdala, while violence was attributed to abnormal electrical activity in the amygdala. Individuals with orbitofrontal injury were found to display antisocial traits (disinhibition, impulsivity, lack of empathy) that justified the diagnosis of “acquired sociopathy”, and some had an increased risk of violent behavior. Cristofori et al. [20] found a correlation between damage to the limbic system, ventromedial prefrontal cortex (vmPFC) and temporal lobes and aggressive behavior. Aggressive behavior was related to frontal lobe dysfunction, as this area is responsible for executive functions and social behavior. This research sample consisted of 112 veterans with TBI and 33 healthy controls (HC) who had no history of brain injury. The two groups were compared for key demographic variables, including age, gender, education and war experiences. The results indicated that the dlPFC and ipTC were required to constrain implicit attitudes toward violence. Lesions involving either area were associated with a more positive implicit attitude toward violence. According to this study, the dlPFC has a pivotal role in downregulating behaviors. Functional neuroimaging studies have shown a correlation between trauma-damaged areas and consequent violent behaviors, but self- and heteroevaluation scales and questionnaires on the construct of aggression and other mental disorders must support these findings. In summary, the areas most involved in violent behaviors are the frontal lobe, prefrontal lobe, orbitofrontal cortex (OFC), compromised white matter integrity and the ventromedial prefrontal ventral cortex (VmPFC). Damage to these areas causes alterations in the processes of inhibition/behavioral initiation, which cause dysfunction of impulse control, moral behavior and emotional regulation.

The structure most involved in violent behavior is the amygdala. A lack of empathy and remorse has been correlated with bilateral damage to the amygdala, while violence has been found to correlate with abnormal electrical activity in the amygdala [41]. Frontal lobe dysfunction in particular has been implicated to explain the actions of some people accused or convicted of violent crimes who apparently fail to inhibit impulsive behaviors [40]. Given the anatomical correlates, it seems clear that beta blockers and/or mood stabilizers should be considered to target restlessness, agitation and aggression [29]. Other agents may occasionally be considered, but these data are more limited [23,33].

### 3.4. Correlation between Younger TBI, Psychiatric Comorbidity and Aggression

Studies have also shown the importance of age at the time of trauma, which could influence deviant behavior.

Adolescence is a risk period for TBI, and TBI is a risk factor for poor mental health and offending behavior [17]. A younger age at the time of the injury has been assessed as an important predictor of aggression following head trauma. Indeed, individuals who were younger at the time of the trauma were more likely to become aggressive [15]. These data could be attributed to the fact that head injuries in childhood and adolescence can lead to poor social communication skills, concomitant externalization behaviors, impulsivity and poor perception of emotions [25,36]. All these consequences can be linked to aggressive and offensive behaviors, including involvement in criminal acts [43]. Children with TBI are more likely to suggest aggressive and avoidant responses to social problems and less likely to generate assertive solutions when compared to children without TBI [37]. Indeed, minors who suffer from TBI have a higher risk of developing a range of internalizing or externalizing disorders linked to an increased risk of criminal acts and violent behavior [11,36].

Children and adolescents with a history of brain injury are four times more likely to develop mental disorders in later life [44]. On the other hand, TBI during childhood and early adolescence significantly increases the risk of criminal offences among people with mental disorders [44]. Generally, patients with a history of head trauma and mental illness often have poor social skills and are poorly equipped to cope with community life, requiring ongoing support [21].

TBI is a risk factor for the subsequent onset of psychiatric illness. Vaughn et al. [5] conducted studies based on the NESARC-III data collected between 2012 and 2013, providing clear results that TBI is highly comorbid with psychiatric disorders, especially violent and non-violent antisocial behaviors. Similarly, Van Reekum [8] investigated the correlation between TBI and changes in mood, personality and behavior. The results revealed high rates of major depression, bipolar affective disorder, generalized anxiety disorder and borderline and avoidant personality disorders. Comorbidity was also high. Interestingly, the absence of episode of psychosis, except those occurring within the context of maniac episode, was observed. These data support the association between TBI and psychiatric disorders, suggesting the need for monitoring, prevention and treatment of psychiatric disorders after TBI.

## 4. Discussion

Our review has found a positive correlation between aggressive behavior and traumatic brain injury. Indeed, violent and aggressive behaviors appear to be related to a history of head trauma, especially if the trauma occurred at an early age. Moreover, stories of TBI seem to have a high incidence of behavioral consequences in individuals, causing personality changes, aggressive behavior and violent and offensive actions. Numerous factors increase the incidence of this correlation, such as environmental, genetic, social and personality factors, temperament, a past history of substance use, the severity of the injury, the age at the time of trauma and poor social support following the injury [18]. Neuroimaging has been used to research the areas that may be the cause of aggressive behavior after trauma by identifying brain injuries and associated damage.

Decreased psychosocial functioning as a result of trauma is a consequence associated with aggressiveness [35]. Head trauma at a young age can be the cause of behavioral consequences in adolescents who engage in offensive and violent attitudes sometimes related to crime, attention disorders, externalizing behaviors, alcohol and substance abuse and conduct disorders. The relationship between head trauma at a young age and the possible development of mental deviance in adulthood, including aggressive behavior and crime, has not yet been fully elucidated. Since adolescence is a risk period for TBI and its potential effects on poor mental health and offending behavior, several recommendations can be made to prevent patients from entering the justice system. Firstly, it could be crucial to identify TBI in adolescents as early as possible and provide appropriate interventions. Prompt diagnosis and treatment could help minimize the potential negative outcomes associated with TBIs, including cognitive and behavioral impairments. Moreover, since adolescents with TBI can still improve their overall functioning and reduce the risk of engaging in offending behavior, they should receive comprehensive medical care, including physical rehabilitation, cognitive therapy and psychological support.

Given the increased risk of mental health issues following a TBI, it is essential to provide appropriate mental health support to adolescents. Access to counseling, therapy and psychiatric services can help address any psychological challenges that may arise, reducing the likelihood of engaging in criminal behavior. 

Social functioning is often a problem in patients with TBI, and this can contribute to a higher risk of entering the justice system. Providing adolescent TBI patients with social support, such as peer support groups, mentorship programs, and community reintegration initiatives, can enhance their social skills, promote positive relationships, and reduce the likelihood of engaging in offending behavior. Specialized educational programs and vocational rehabilitation can also help them to acquire skills, increase self-esteem, and improve their employment prospects, reducing the risk of criminal involvement.

Among the limitations of this study, it should be mentioned that the review was conducted on studies spanning a 15-year period, and there may have been updates on the topic since then. Therefore, it might be interesting in future studies to use this 15-year period as a reference point to see what has changed from that time to the present.

Systematically and thoroughly studying the relationship between TBIs and behavioral disorders by assessing the incidence of all other factors could be the key to finding the predictors of violent behavior and taking timely action. Another factor that can be assessed in future studies may be the emotional experience related to possible trauma suffered in childhood and beyond, which may have influenced cognitive degeneration, causing deviant personality traits related to crime. Furthermore, development of these issues could lead to the development of increasingly specific and individualized treatments based on the specific needs and circumstances of a patient. Collaborative efforts among healthcare professionals, educators, social workers and the justice system can facilitate a comprehensive and holistic approach to supporting adolescents with TBIs and reducing their risk of entering the justice system.

## Figures and Tables

**Table 1:** Summary of studies included in the research.

Study	Aim	Method	Outcome
Baguley et al. (2006) [15]	Evaluate the occurrence and factors influencing aggressive conduct in individuals who have experienced traumatic brain injury (TBI) during three different time points: 6 months, 24 months and 60 months after their discharge from medical care.	Data were collected within a specialized brain injury rehabilitation hospital Service	During each of the follow-up periods, approximately 25% of the participants were identified as displaying aggressive behavior. This group showed connections with depression, concurrent traumatic complaints, being of a younger age at the time of injury and experiencing low life satisfaction. These associations were found to be more relevant than injury-related factors or demographic information. Notably, depression emerged as the most significant factor linked to aggressive behavior.
Veeh et al. (2018) [16]	Investigate whether temperament mediates the relationship between TBI and violent behavior within incarcerated youth.	An evaluation was conducted encompassing traumatic brain injury (TBI), temperament, childhood trauma, substance use, mental health conditions and various demographic factors.	The interaction between effortful control and negative emotionality seems to play a crucial role as a mechanism that influences the connection between traumatic brain injury (TBI) and violent behavior.
Williams et al. (2010) [17]	Investigate whether self-reported traumatic brain injury (TBI) in male young offenders is a risk factor for reoffending, poor mental health, and violence.	Data were collected through self-reports and interviews with male young offenders who reported a history of traumatic brain injury.	Male young offenders with a history of TBI exhibited an increased risk of reoffending, poor mental health, and violent behavior.
Colantonio et al. (2007) [14]	Examine brain injury prevalence in a forensic psychiatry population.	Medical records of forensic psychiatry patients were examined to identify the prevalence of brain injuries.	A significant proportion of the forensic psychiatry patients had evidence of brain injuries.
Darby, R.R. (2018) [18]	Investigate neuroimaging abnormalities in neurological patients with criminal behavior.	Neuroimaging data from neurological patients displaying criminal behavior were analyzed to identify any abnormalities.	Neuroimaging analysis revealed abnormalities in neurological patients exhibiting criminal behavior.
Glenn & Raine (2014) [19]	Examine the implications of neurocriminology for the punishment, prediction and prevention of criminal behavior.	The implications of neurocriminology for criminal behavior were analyzed, focusing on punishment, prediction and prevention.	The study discussed the potential applications of neurocriminology in dealing with criminal behavior, including its use in punishment, prediction and prevention.
Cristofori et al. (2016) [20]	Investigate brain regions influencing implicit violent attitudes using a lesion-mapping study.	A lesion-mapping study was conducted to identify brain regions influencing implicit violent attitudes.	The study identified specific brain regions that played a role in influencing implicit violent attitudes.
Timonen et al. (2002) [21]	Assess the association between preceding traumatic brain injury and mental disorders, alcoholism, and criminality using the Northern Finland 1966 Birth Cohort Study.	The Northern Finland 1966 Birth Cohort Study data were analyzed to assess the association between preceding traumatic brain injury and mental disorders, alcoholism and criminality.	Preceding traumatic brain injury was associated with an increased risk of mental disorders, alcoholism and criminal behavior.
Slaughter et al. (2003) [22]	Examine the prevalence of traumatic brain injury (TBI) and its association with neuropsychological functioning and psychiatric disorders in a county jail population.	TBI prevalence, neuropsychological functioning and psychiatric disorders were assessed in a county jail population.	TBI was prevalent in the county jail population and associated with neuropsychological impairments and psychiatric disorders.
Deb et al. (2020) [23]	Investigate the feasibility of using risperidone as a treatment for aggression following traumatic brain injury.	A randomized controlled trial was conducted to compare risperidone with a placebo for aggression in traumatic brain injury patients.	The feasibility trial suggested that risperidone may be a potential treatment for aggression after traumatic brain injury.
Epstein et al. (2016) [24]	Examine orbitofrontal cortical thinning and its relationship with aggression in patients with mild traumatic brain injury.	The study utilized neuroimaging techniques to investigate orbitofrontal cortical thinning in mild traumatic brain injury patients exhibiting aggression.	Patients with mild traumatic brain injury and aggression showed orbitofrontal cortical thinning in neuroimaging scans.
Vaughn et al. (2014) [25]	Identify correlates of traumatic brain injury (TBI) in a multi-site study of juvenile offenders.	A multi-site study was conducted to identify the correlates of TBI among juvenile offenders.	The study identified various correlates of traumatic brain injury in juvenile offenders.
Dailey et al. (2018) [26]	Investigate elevated aggression and reduced white matter integrity in mild traumatic brain injury patients.	A diffusion tensor imaging (DTI) study was conducted to assess white matter integrity in mild traumatic brain injury patients with elevated aggression.	Mild traumatic brain injury patients with elevated aggression showed reduced white matter integrity in DTI scans.
Elbogen et al. (2015) [27]	Examine longitudinal predictors of criminal arrest after traumatic brain injury using the Traumatic Brain Injury Model System National Database.	The study utilized data from the Traumatic Brain Injury Model System National Database to explore longitudinal predictors of criminal arrest after traumatic brain injury.	The study identified longitudinal predictors associated with criminal arrest after traumatic brain injury.
Gordon et al. (2017) [28]	Investigate the relationship between traumatic brain injury (TBI) and criminality in juvenile offenders.	The study examined the relationship between TBI and criminal behavior in juvenile offenders.	A relationship between traumatic brain injury (TBI) and criminal behavior was found in juvenile offenders.
Robert, S. (2020) [29]	Explore the association between traumatic brain injury (TBI) and mood disorders.	The study investigated the association between TBI and mood disorders.	The study found an association between traumatic brain injury (TBI) and mood disorders.
Jackson et al. (2017) [30]	Examine the relationship between early childhood head injury and later life criminal behavior using a longitudinal cohort study.	A longitudinal cohort study was conducted to investigate the relationship between early childhood head injury and later life criminal behavior.	The study revealed a relationship between early childhood head injury and later life criminal behavior.
Tateno et al. (2003) [31]	Identify clinical correlates of aggressive behavior after traumatic brain injury.	The study examined clinical correlates of aggressive behavior in patients with traumatic brain injury.	The study identified clinical correlates associated with aggressive behavior after traumatic brain injury.
Miles et al. (2021) [32]	Investigate the evolution of irritability, anger and aggression after traumatic brain injury and identify subgroups with predictive factors.	The study analyzed the evolution of irritability, anger and aggression after traumatic brain injury, identifying subgroups with predictive factors.	The study identified subgroups with specific predictive factors for the evolution of irritability, anger and aggression after traumatic brain injury.
Hammond et al. (2021) [33]	Assess the efficacy of carbamazepine for treating irritability and aggression after traumatic brain injury in a randomized, placebo-controlled study.	A randomized, placebo-controlled study was conducted to assess the efficacy of carbamazepine for treating irritability and aggression in traumatic brain injury patients.	Carbamazepine showed promise for treating irritability and aggression after traumatic brain injury in the study.
Darby et al. (2018) [34]	Investigate lesion network localization of criminal behavior.	The study used lesion network localization techniques to investigate criminal behavior localization in patients with brain lesions.	Lesion network localization revealed specific brain lesion locations associated with criminal behavior.
Roy et al. (2017) [35]	Examine the prevalence and correlates of aggression at six months and one year after first-time traumatic brain injury.	The study investigated the prevalence and correlates of aggression at six months and one year after first-time traumatic brain injury.	The study found prevalence rates and correlates of aggression at six months and one year post-traumatic brain injury.
McCormick et al. (2021) [36]	Investigate mild traumatic brain injury as a predictor of youth internalizing and externalizing psychopathology using a longitudinal study.	A longitudinal study was conducted to investigate mild traumatic brain injury as a predictor of youth internalizing and externalizing psychopathology.	Mild traumatic brain injury was found to be a predictor of youth internalizing and externalizing psychopathology.
Ganesalingam et al. (2007) [37]	Examine self-regulation as a mediator of the effects of childhood traumatic brain injury on social and behavioral functioning.	The study investigated self-regulation as a mediator of the effects of childhood traumatic brain injury on social and behavioral functioning.	Self-regulation mediated the effects of childhood traumatic brain injury on social and behavioral functioning.
Moore et al. (2014) [38]	Examine the relationship between traumatic brain injury (TBI), mental health, substance use and criminal offense among incarcerated young people.	The study investigated the relationship between TBI, mental health, substance use and criminal offense in incarcerated young people. Data were collected and analyzed to assess the association between these factors.	Incarcerated young people with TBI were found to have higher rates of mental health issues, substance use, and offending behaviors.
Van Reekum et al. (1996) [8]	Explore psychiatric disorders after traumatic brain injury.	The study examined psychiatric disorders following traumatic brain injury.	Psychiatric disorders were found to be prevalent after traumatic brain injury.
Vaughn et al. (2019) [5]	Investigate the relationship between traumatic brain injury (TBI) and psychiatric comorbidity in the United States.	Data from the United States were analyzed to study the relationship between TBI and psychiatric comorbidity.	A relationship was observed between traumatic brain injury (TBI) and psychiatric comorbidity in the United States.

## Data Availability

Not applicable.
